# Effects of Diet, Nutrition, and Exercise in Children With Autism and Autism Spectrum Disorder: A Literature Review

**DOI:** 10.7759/cureus.12222

**Published:** 2020-12-22

**Authors:** Shriya Doreswamy, Anam Bashir, Jesus E Guarecuco, Simmy Lahori, Ayesha Baig, Lakshmi Rekha Narra, Pinal Patel, Stacey E Heindl

**Affiliations:** 1 Internal Medicine, California Institute of Behavioral Neurosciences and Psychology, Fairfield, USA; 2 Otorhinolaryngology, Vydehi Institute of Medical Sciences and Research Centre, Bangalore, IND; 3 Neuroscience and Psychology, California Institute of Behavioral Neurosciences and Psychology, Fairfield, USA; 4 Medicine, Pramukhswami Medical College, Karamsad, Anand, IND; 5 Medicine, California Institute of Behavioral Neurosciences and Psychology, Fairfield, USA; 6 Anesthesiology, California Institute of Behavioral Neurosciences and Psychology, Fairfield, USA; 7 Internal Medicine, Avalon University School of Medicine, Willemstad, CUW

**Keywords:** autism, autism spectrum disorder, diet, nutrition and exercise, pediatric assessment, metabolism, supplements, gluten-free diet, applied behavioral analysis (aba), complementary and alternative medicine (cam)

## Abstract

Diet and nutrition play an important and essential role in everyone's life. It helps build a healthy body and a strong mind. We know that food rich in nutrients can remove toxins from the body, make an excellent immune system, curb hunger, and prevent obesity. Obesity is one of the most concerning, alarming, and fastest-growing pandemics. It affects not only adults but also adolescents and children. The disease's early-onset calls for prompt attention to control the physical, psychological, financial, and social burden it creates. Children with autism and autism spectrum disorders (ASDs) are commonly affected by eating disorders. Their preference for energy-dense food with low nutrition can alter their metabolism, leading to the accumulation of oxidative radicals, causing them to deteriorate mentally and physically. Although dieting and losing weight are now commonly seen in the general population, it has become hard to bring awareness to children with special needs about diet, nutrition, and obesity. Despite efforts, parents of such children usually cannot help control the eating because tantrums and behavioral problems are common. It is now imperative for doctors and parents to work alongside nutritionists and dieticians to help these children eat healthy to be fit and improve the quality of life.

## Introduction and background

Uncontrolled and unhealthy eating habits commonly lead to physical changes such as being overweight and obesity in the population. These body changes alter our well-being, leading to multiple health issues by causing high cholesterol, high triglycerides, high blood sugar, and electrolyte imbalance. Comorbidities like diabetes mellitus (DM), coronary artery disease (CAD), stroke, and many more disorders become common [1].

Persons with uncontrolled eating habits may have undiagnosed eating disorders (ED). Studies indicate that ED may be one of the significant contributors to comorbidities [2]. Children with neurodevelopmental disorders such as autism and autism spectrum disorders (ASDs) are the commonest population to manifest with ED when compared to the general population [2]. Recent studies indicate that up to three percent of children or at least one in 68 children in the USA may have autism and ASD [3,4]. Children with autism and ASD have reduced social interactions, no eye contact, repetitive stereotypic sensory and motor behaviors, and reduced to no communication with others [4]. Reports also indicate that individuals with autism and ASD are less likely to participate in any physical activity, leading to overweight and obesity [5]. These children are noted to be picky eaters and prefer junk food: calorie-dense, carbohydrate-dense with high sodium, and less nutrition [1]. 

It is our duty as doctors and parents to nourish the culture of eating healthy as children with special needs may not completely understand the concept and the benefits of healthy and nutritious food [4]. Food rich in macronutrients such as proteins, fats, and micronutrients such as vitamins and minerals, acts as antioxidants and improves these children's better functioning [3]. Interestingly, studies from Catassi et al. suggesting a gluten-free diet and Żarnowska et al. proposing a carbohydrate-free ketone diet (KD) have shown tremendous improvement in the behavior and cognitive skills of these children [6,7].

The decreased physical activities and increased erratic food habits can also lead to long-term health imbalance and societal costs of overweight and obesity in this population [8]. The health burden does not limit to just medication bills but includes their nutritionist, dietician, and pediatrician fees. In their study, Criado et al. mention that the societal costs include special education, occupational therapy, speech therapy, applied behavioral analysis (ABA) therapy in these children, which are not covered by the insurance [1]. The study also mentions three other studies that talk about pharmacotherapy, where medications like antipsychotics used in treating ASD and autism can further be one of the causes of unintentional weight gain due to side effects [1]. 

Although pediatricians measure height, weight, and body mass index (BMI) on every well-child visit, very little progress is made to curb obesity and overweight and promote healthy food habits. Many studies also indicate that parents of children with autism and ASD have no formal education, belong to high socioeconomic status, have reduced sleep and affective problems that significantly contribute to their condition [1,5]. Reports indicate that at least 30 percent of the ASD and autistic children fall under the obese category [3,4]. We know unhealthy eating habits in children get carried forward to adolescence and adulthood, leading to obesity that reduces life quality and causes comorbidities. A genetic link between autism and ASD with food habits, drug efficacy, and potency needs research in these children. It is necessary to establish a streamlining of nutrition and physical activity for children with autism and ASD. In this paper, we review articles and arrive at collective data to help primary care physicians, pediatricians, parents, and everyone involved with the treatment of autism and ASD children to improve their lifestyle. 

## Review

Methods

On PubMed, we used the medical subject heading (MeSH) words "Autism," "Autism Spectrum Disorders," and "Obesity" to search for articles between the years 2000 and 2020 initially. Since the data was insufficient, we extended the number of years by another 20 years to 1980. This search yielded 69 papers that were all in English. We applied the population's inclusion criteria as under 18 years of age, all human studies, and available for free full-text articles. It resulted in 30 papers, including literature reviews, case-control studies, meta-analysis studies, and surveys. We used the same words on Google Scholar, which yielded us 200 results, and with all the inclusion criteria mentioned above, we got 11 results that were of importance to us. We also referred to the Centers for Disease Control (CDC) website, which yielded us one paper. For the nutritional studies on autistic and ASD children, we used the words "Diet" and "Nutrition" in "Autism and ASD" on MeSH, which yielded us 345 articles, of which only 156 had free full-text articles in the same time frame of 40 years. We chose papers that were most relevant to our topic of interest, and we were left with 10 studies. In our final report, we used 27 articles to remove all the duplicate papers and essays.

Results

Using the inclusion criteria of obesity, nutrition, and diet in human studies under 18 years of age, applying filtration criteria of papers in the past 40 years in the English language only, we were left with 27 studies that were most relevant to our article and hence included them. Through these papers, we were able to determine the relationship between diet and nutrition in children with autism and ASD and improve their functionality.

Discussion

Pediatric Assessment

Every well-child visit mandates an evaluation of obesity. These evaluations are done mainly by pediatricians or primary care providers. The CDC defines BMI as an individual that falls above the 95th percentile of weight on the scale [4]. It is the same in all children, including autistic and ASD children. Many pediatricians do not receive training to manage overweight and obesity, let alone managing these conditions in autistic or ASD children [3]. Hence, they usually refer these children to a developmental pediatrician or a dietician for exquisite counseling and management [4]. They also associate this unmanageability with the intelligence quotient (IQ) of the children [9]. The CDC puts forth that children with ASD have varying IQs, as can be seen in Table 1 [10] 

**Table 1:** Classification of intelligence quotient (IQ)

IQ	Level of Intelligence
<70	Intellectual disability
71-85	Borderline range
>85	Average or higher range

In the last few years, developmental pediatricians diagnose autism as early as 18 months [4]. Early diagnosis and early interventions through therapies such as speech, occupational, and ABA therapy help them improve intellectually and teach them life skills that help them interact with peers and improve their sensory and motor skills, including table manners [11]. Although these interventions promise good behavioral outcomes, it is mandatory to organize their diet to control obesity. 

Streamlining Nutrition and Diet

Curtin et al. discuss in their study that it is essential for children to develop clean eating habits and incorporate family practices like sitting down at the table for a meal, not watching gadgets or television, having a family conversation, and learn to eat on their own [11]. Children with autism and ASD find it most challenging to adapt to new food and new rules around food [12]. Hill et al. mentioned in their studies that autistic and ASD children prefer energy-dense, nutrient-deficient foods and reject fruits, vegetables, and whole grains [5]. The autistic and ASD children are "picky eaters" with preference to only certain food types, tastes, and textures; hence selective eating becomes a huge problem for parents and everyone dealing with them [12]. Parents must teach good eating habits in these children right from when they are weaned from mother's milk to help curb these problems.

Along with the above, a specialized diet in place and restricted food preferences, introduction to a new food or physical activity, can frequently cause disruptive behavior that ultimately leads parents to budge to their demands [12]. Hence, new foods should be introduced to these children in phases to ensure familiarity with the food's taste and texture [13]. While all these studies only mention the intervention, it does not describe the methods, timing, or the phases of the introduction of food that we can do. 

Alteration in Metabolism

A study by Żarnowska et al. in 2018 conducted a therapeutic use of carbohydrate-restricted diet/KD in an autistic child that concluded significant clinical outcomes like better attention span, communication skills, reduced fear, anxiety, and emotional disturbances. They also attributed the personal and behavioral changes to reduced gene expression in the mitochondria and reduced activity in mitochondria's electron chain [7]. Studies by Napoli et al. in 2014 and Cheng et al. in 2017 showed that individuals on a carbohydrate-less diet became ketone dependent, decreasing mitochondrial function and reducing their energy requirements to show significant behavior improvement [14,15]. Although this research has generated a powerful insight into the mechanism, it is necessary to have further studies to prove the research. 

Vitamins and Mineral Supplements

Vitamins and mineral supplements are essential in children with autism and ASD as they are considered highly beneficial. Many of these vitamins and minerals act as co-enzymes and neurotransmitters for numerous biochemical enzymatic reactions in our bodies. Adams et al. did a randomized control study for three months that deciphered that low levels or absence of vitamins and minerals can result in impaired metabolic functioning [16]. The study concluded that while the supplements improved biotin, oxidative stress, glutathione, methylation, adenosine triphosphate (ATP), the reduced form of nicotinamide adenine dinucleotide phosphate (NADPH), and sulfate, they also reduced the hyperactivity, tantrum, and overall reception in the language with fewer side effects [16]. Buie et al., Horvath et al., and Ashwood et al. conducted studies that proved that a Parent Global Impression-Revised (PGI-R) score improved significantly [17-19]. A PGI-R score is an indicator of psychosocial impairment in children with special needs, including various factors such as pain and hunger that can be measured [17]. It has high sensitivity, specificity, is highly consistent, reliable, and valid as it determines the effectiveness of all the therapies given to autistic children [20]. These studies' limitations were that they were done on small sample size and needed in-depth research.

Gluten-Free Diet

Research on diet and nutrition in autistic and ASD kids has substantially increased in the past 20 years, specifically to study hyperactivity and attention. Catassi et al. and Cruchet et al. conducted two different studies on nonceliac gluten sensitivity-based, gluten-free diet trials in 2016 and 2013, respectively, on autistic and ASD kids [6,21]. The gluten-free diet type of test was first done in the 1980s and recently rediscovered, which hypothesized a possible increase in peptide formation due to incomplete and improper breakdown of food containing gluten and casein [6,21]. A "leaky gut" phenomenon seen in autism and ASD could cause these peptides to cross the blood-brain barrier affecting the endogenous opiate neurotransmission mechanisms, and the removal of these so-called toxins helps the children control their actions and emotions [6,21]. Another study by Robertson et al. compared original research done by Lau et al. regarding intestinal permeation and did not find any intestinal permeability changes, as mentioned by the latter [22,23]. Despite all these studies and the popularity of trying different diet plans for these kids, there is more to prove these effects.

Complementary and Alternative Medicine

Zuckerman et al. mention in their studies that many parents have also explored the options of treating children with chiropractors and dietary supplements, including digestive enzymes, microminerals, no sugar diet, probiotics, yielding inconclusive results [24]. Research by Hofer et al. uses food supplements such as omega-3 fatty acids, folinic acid, L-carnosine, tetrahydrobiopterin, N-acetylcysteine, oxytocin, dimethylglycine to see possible changes in these children [25]. Although these do not give great results, the research is not entirely false either, the reason being, they have all been studied in a tiny population for a brief period. We need more research on this aspect. 

Medications

Melatonin is one of the most familiar drugs used in autistic and ASD kids to treat sleep disorders and disturbances and is one of the well-tolerated drugs giving good results. The only drawback is that it is being studied in a tiny population among these kids, as many parents do not want to provide these medications with a fear of severe side effects [25]. Many drugs like antipsychotics, stimulants, and antiepileptics used in treating autism and ASD children play a significant role in gaining weight [5]. In their study, Egan et al. mention no relationship between medications and weight gain, unlike the many other studies in the past [3]. 

Microbiota

In recent times, the microbiota has become an essential topic of interest. They are necessary for our intestine, unblemished skin, or sound mental and physical health. In 2017, a study by Kang et al. linked gut microbiome to abnormal metabolites and behavior, showing that the transplant of microbiota causes a significant improvement in constipation symptoms, diarrhea, indigestion, abdominal pain, and behavior [26]. For this reason, many parents fed the kids fresh homemade yogurt at least two times a day. Many studies conducted in the past have also concluded that using probiotics is safe and a promising treatment for children with autism and ASD. 

**Figure 1:**
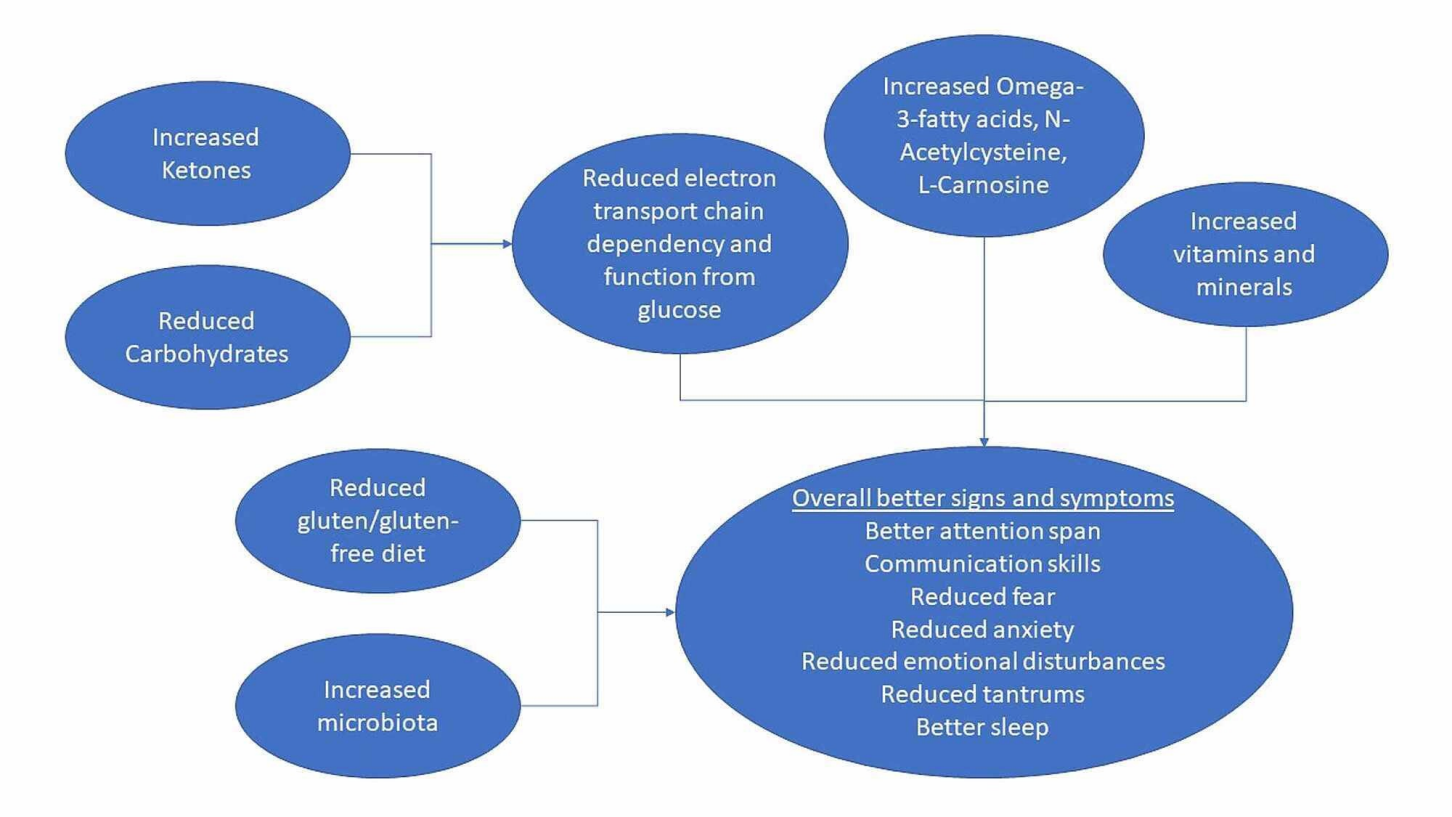
Dietary regulation of metabolism in autistic and ASD children ASD: Autism spectrum disorder

Table 2 summarizes the studies mentioned in our discussion. A summary of dietary regulation of metabolism in autistic and ASD children to improve their lifestyle is in Figure 1. 

**Table 2:** Synopsis of metabolic regulation from past studies ASD: Autism spectrum disorder; PGI-R: Patient Global Impression-Revised

Author	Year	Sample Size (N)	Conclusion
Żarnowska et al. [7]	2018	35 and a single case study	Concluded that the ketone diet shows tremendous improvement in behavior, eating habits, and tantrums
Nadon et al. [13]	2010 and 2011	95 cases, compared autistic children with non-autistic siblings from the same family.	Identified sensory problems concerning food and eating habits. Concluded that sensory habits are completely different and can be haywire in these children.
Napoli et al. [14]	2014	N/A	A ketogenic diet reduces mitochondrial gene expression helping improve symptoms.
Cheng et al. [15]	2017	N/A	A ketogenic diet reduces mitochondrial gene expression, and hence there is a significant improvement in behavior, hunger, and other major autistic symptoms.
Adams et al. [16]	2011	141	Vitamins and minerals have good effects on ASD and autism, improved PGI-R subscales, tantrums, receptive language.
Cruchet et al. [21]	2016	N/A	Nonceliac gluten sensitivity diet and vegetarian diet improve the behavioral outcome.
Zuckerman et al. [24]	2015	376	Complementary and alternative medicines worked better for most children. Reduced medication and melatonin worked great with the least side effects and improvement in sleep.
Jones et al. [27]	2017	35	Increased socialization, motor skills, and physical activity regulates hunger and changes social-emotional and developmental functioning.

Therapy

As we mentioned earlier, it is imperative to recognize the signs and symptoms first and intervene early for best results. Early intervention also ensures adequate exposure to different people, various environments, and situations, identifying possible stressors, and working on them. ASD and autistic children need to mingle with typically developing children as their company helps them grow emotionally and socially, improve physical health, broaden their outlook towards life, increase interests in different aspects, and strengthen motor skills [27]. Exercising also boosts metabolism and can regulate hunger and satiety centers for food [9]. Therapies such as occupational, speech, and ABA help deal with all the sensitivities and activities necessary to get back to the "normal" or "typical" group while creating a conducive environment for these children to improve their quality of life [12]. The significant drawbacks are that insurance does not cover many of these therapies and can cause a tremendous burden on the caregivers' pockets. 

Limitations

Our study has aimed to put forth the connection that children with autism and ASD have with food habits. Although studies show significant changes in behavior, emotion, and cognitive skills in autistic and ASD children due to small changes in food habits, there is no concrete evidence to prove the same. Most of the studies are done only on a small population of autistic children. The data we collected focuses on a population mostly in the Western world, including developed countries, and does not include third-world countries. A few other limitations to this topic are that few articles are available, limited knowledge among the primary caregivers and pediatricians, and, most of all, little reporting and treatment of such children that significantly affect the results. 

## Conclusions

Challenging situations promote thought-provoking questions, which further lead to in-depth research and analysis. One such topic is autism. Developmental pediatricians and doctors treating adult autistic and ASD cases commonly say that every autistic person is unique. Everyone has different signs and symptoms or challenges. To better understand the patients, we first need to train the providers. We should survey the pediatricians and parents to know more about their challenges, particularly food and food habits. Changes and interventions should be brought about in children as early as possible to give them a better quality of life. Just like food, physical activity plays an equally important role in managing these children. Physical activity helps in weight management and helps release their stresses along with providing social interactions. It also releases the 'feel-good hormones' or the endorphins that help in faster healing. A trial and error of diets is a must to see what suits them best. The medications need to be titrated and monitored as one medicine can act differently in different autistic children and autistic adults. For all these to function correctly, the providers, therapists, dieticians, parents, and everyone involved need to work hand in hand. To draw conclusive results, the federal government must give more grants, plenty of research, and, most of all, all expenditure needs to be covered by the insurance. Do we conclude by wondering if obesity is linked to genetic causes, and will we manage it in these children? Only further studies and research will tell.
